# THE DOUBLE BURDEN OF CIRRHOSIS AND ACUTE PANCREATITIS: AMPLIFIED RISKS FOR NUTRITIONAL AND GASTROINTESTINAL COMPLICATIONS

**DOI:** 10.1590/S0004-2803.24612025105

**Published:** 2026-05-18

**Authors:** Chidera N ONWUZO, Kikunlore Elijah ODUNSANYA, FNU ALVINA, Rashid Abdel RAZEQ, Kojo-Frimpong B. AWUAH, Somtochukwu ONWUZO

**Affiliations:** 1 SUNY Upstate Medical University, Resident Physician, Syracuse, NY, USA.; 2 Allegheny General Health, Fellow Physician, Pittsburgh, PA, USA.; 3 Cleveland Clinic Fairview Hospital, Resident Physician, Cleveland, OH, USA.; 4 Olabisi Onabanjo University Teaching Hospital, Hematology Department, Ogun State, Nigeria.

**Keywords:** Cirrhosis, acute pancreatitis, nutritional complications, protein-calorie malnutrition, Ileus, small bowel obstruction, pseudocyst, total parenteral nutrition (TPN), percutaneous endoscopic gastrostomy (PEG), TriNetX, Cirrose, pancreatite aguda, complicações nutricionais, desnutrição protéico-calórica, íleo, obstrução do intestino delgado, pseudocisto, nutrição parenteral total (NPT), gastrostomia endoscópica percutânea (GEP), TriNetX

## Abstract

**Background:**

Cirrhosis and acute pancreatitis (AP) are significant causes of morbidity and mortality, and their coexistence magnifies the risk of systemic complications. Patients with both conditions are particularly vulnerable to adverse outcomes, including gastrointestinal and nutritional challenges. This study examines the impact of cirrhosis on 1-year outcomes in AP patients, focusing on pseudocyst formation, protein-calorie malnutrition, ileus, small bowel obstruction, and the need for nutritional interventions such as total parenteral nutrition (TPN) or percutaneous endoscopic gastrostomy (PEG).

**Methods:**

A retrospective cohort analysis was conducted using TriNetX database. Patients with AP were identified and divided into two cohorts: those with cirrhosis (Cohort 1) and those without cirrhosis (Cohort 2). Propensity score matching balanced baseline characteristics, resulting in 26,160 patients in each cohort. Outcomes were tracked over one year, including pseudocyst formation, ileus, small bowel obstruction, malnutrition, and nutritional interventions. Kaplan-Meier survival estimates and hazard ratios with 95% confidence intervals assessed risk differences between cohorts.

**Results:**

Cirrhosis significantly worsened outcomes compared to non-cirrhosis. Protein-calorie malnutrition was twice as common (*P*<0,001), affecting 5.2% of cirrhotic patients versus 2.6% of non-cirrhotic patients. Risks for ileus (*P*<0,001) and small bowel obstruction (*P*<0,001) were also significantly higher, with incidences of 2.7% and 1.7%, respectively, in cirrhotic patients. The need for TPN was more frequent in the cirrhosis cohort (*P*<0,001), affecting 1.9% compared to 1.2%. Conversely, pseudocyst formation was less frequent in cirrhotic patients (*P*=0,018), with an incidence of 2.3% versus 2.8%. PEG insertion and abdominal compartment syndrome showed no significant differences.

**Conclusion:**

Cirrhosis amplifies the risk of nutritional deficiencies, ileus, and small bowel obstruction in AP patients, emphasizing the need for proactive, multidisciplinary management strategies for this high-risk population. The reduced incidence of pseudocyst formation may reflect altered disease progression in cirrhosis.

## HIGHLIGHTS

• This retrospective cohort study evaluated the impact of cirrhosis on 1-year gastrointestinal and nutritional outcomes in patients with acute pancreatitis (AP) using the TriNetX database.

• Compared with non-cirrhotic AP patients, those with cirrhosis had significantly higher risks of protein-calorie malnutrition, ileus, small bowel obstruction, and need for intensive nutritional support, including total parenteral nutrition (TPN).

• Pseudocyst formation was less frequent in cirrhotic AP patients, while rates of PEG insertion and abdominal compartment syndrome were similar, suggesting a distinct pattern of disease evolution in the setting of cirrhosis.

## RESUMO

**Contexto:**

A cirrose e a pancreatite aguda (PA) são causas importantes de morbidade e mortalidade, e a sua coexistência amplifica o risco de complicações sistêmicas. Pacientes com ambas as condições são particularmente vulneráveis a desfechos adversos, incluindo desafios gastrointestinais e nutricionais.

**Objetivo:**

Este estudo avalia o impacto da cirrose nos desfechos em 1 ano em pacientes com PA, com foco na formação de pseudocisto, desnutrição proteico-calórica, íleo, obstrução de intestino delgado e na necessidade de intervenções nutricionais, como nutrição parenteral total (NPT) ou gastrostomia endoscópica percutânea (GEP).

**Métodos:**

Foi realizada uma análise retrospectiva de coorte utilizando a base de dados TriNetX. Pacientes com PA foram identificados e divididos em duas coortes: aqueles com cirrose (Coorte 1) e aqueles sem cirrose (Coorte 2). O pareamento por escore de propensão foi utilizado para balancear as características basais, resultando em 26.160 pacientes em cada coorte. Os desfechos foram acompanhados ao longo de um ano, incluindo formação de pseudocisto, íleo, obstrução de intestino delgado, desnutrição e intervenções nutricionais. Estimativas de sobrevivência de Kaplan-Meier e razões de risco (hazard ratios, HR) com intervalos de confiança de 95% (IC95%) foram utilizadas para avaliar as diferenças de risco entre as coortes.

**Resultado:**

A cirrose piorou significativamente os desfechos em comparação com a ausência de cirrose. A desnutrição proteico-calórica foi duas vezes mais comum (*P*<0.001), acometendo 5,2% dos pacientes cirróticos versus 2,6% dos pacientes não cirróticos. Os riscos de íleo (*P*<0.001) e de obstrução de intestino delgado (*P*<0.001) também foram significativamente maiores, com incidências de 2,7% e 1,7%, respectivamente, nos pacientes cirróticos. A necessidade de NPT foi mais frequente na coorte com cirrose (*P*<0.001), acometendo 1,9% em comparação com 1,2%. Por outro lado, a formação de pseudocisto foi menos frequente em pacientes cirróticos (*P*=0.018), com incidência de 2,3% versus 2,8%. A realização de GEP e a ocorrência de síndrome compartimental abdominal não apresentaram diferenças significativas.

**Conclusão:**

A cirrose amplifica o risco de deficiências nutricionais, íleo e obstrução de intestino delgado em pacientes com PA, ressaltando a necessidade de estratégias de manejo proativas e multidisciplinares para essa população de alto risco. A menor incidência de formação de pseudocisto pode refletir uma progressão de doença alterada na cirrose.

## INTRODUCTION

Acute pancreatitis (AP) is a severe inflammatory disease characterized by the autodigestion of the pancreas due to premature activation of digestive enzymes^1^. The incidence of AP has been rising globally, largely attributed to modern lifestyle factors such as increased alcohol consumption, diets high in fats, obesity, and gallstone disease^2^. Despite this increasing incidence, mortality rates have declined in recent years due to improved management^3^. The revised Atlanta classification categorizes AP into mild, moderately severe, and severe forms, with mild cases comprising 75-80% of all patients^3^ and associated with less than 1% mortality and shorter hospital stays. In contrast, severe AP can have mortality rates as high as 20-40%^4^. The pathophysiology of AP involves complex inflammatory processes that increase vascular permeability, resulting in capillary leakage, third-spacing of fluids, intravascular volume depletion, hypotension, shock, and multi-organ failure^5^.

Liver cirrhosis (LC), the end-stage of chronic liver disease (CLD), is marked by progressive fibrosis, portal hypertension, and impaired hepatic function^6^. Globally, LC contributes significantly to morbidity and mortality, particularly in regions with high prevalence of hepatitis, alcohol abuse, or non-alcoholic fatty liver disease^7^. Cirrhosis is associated with immune dysfunction, altered coagulation, and compromised organ perfusion, which can worsen outcomes in acute systemic illnesses^8^.

Both AP and LC impose considerable healthcare burdens due to their increasing prevalence. Notably, they share many overlapping risk factors such as alcohol abuse and obesity resulting in frequent coexistence, particularly in alcohol-related cases. Epidemiological studies, including a 20-year Finnish study, have documented parallel rises in the incidence of alcoholic AP and cirrhosis, corresponding with alcohol consumption trends^9^. This overlap underscores the intertwined nature of these diseases.

While both AP and LC independently lead to nutritional and gastrointestinal complications, there is limited data exploring how cirrhosis amplifies these risks in patients with acute pancreatitis. Most studies tend to examine these conditions separately without investigating their combined impact.

This study aims to address this gap by evaluating the amplified risk of nutritional and gastrointestinal outcomes in AP patients with cirrhosis. Specifically, it focuses on complications such as pancreatic pseudocyst formation, protein-calorie malnutrition, ileus, small bowel obstruction, and the increased need for nutritional interventions. Understanding this relationship is crucial for optimizing management and improving prognosis.

## METHODS

We performed a retrospective cohort study of adults with acute pancreatitis utilizing patient data from the TrinetX US Collaborative Research Network, a large multisystem database with over 100 million patients. This retrospective cohort study included adults diagnosed with cirrhosis of unspecified etiology and acute pancreatitis (AP), matched to patients of the same age group with AP but without cirrhosis. Patients with diagnosis of AP were identified using ICD-10 codes. Propensity score matching was performed to ensure balanced demographic and clinical characteristics between the cirrhotic and non-cirrhotic AP cohorts. Propensity Score Matching (PSM) is a statistical technique for observational studies to reduce selection bias by estimating the causal effect of a treatment, policy, or intervention by creating a comparable control group. It works by first calculating a propensity score, the probability of a subject receiving the treatment based on their observed pre-treatment characteristics and then matching subjects in the treatment group to subjects in the control group with similar propensity scores. This process helps to balance the groups on observed characteristics, allowing for a more reliable comparison of outcomes.

The diagnosis of Acute Pancreatitis was determined using the International Classification of Diseases, Tenth Revision, Clinical Modification (ICD-10-CM). For data originally coded in ICD-9-CM, the study utilized TriNetX’s mapping to convert it to ICD-10-CM, using general equivalence mappings and custom algorithms to ensure accuracy. The patient population was segregated into two groups: AP patients with cirrhosis and AP patients without cirrhosis.

The diagnosis of Acute Pancreatitis was determined using the International Classification of Diseases, Tenth Revision, Clinical Modification (ICD-10-CM). For data originally coded in ICD-9-CM, the study utilized TriNetX’s mapping to convert it to ICD-10-CM, using general equivalence mappings and custom algorithms to ensure accuracy. The patient population was segregated into two groups: AP patients with cirrhosis and AP patients without cirrhosis.

We extracted data from the TriNetX global collaborative network, a federated research platform aggregating electronic health records from a diverse range of healthcare organizations across 30 countries worldwide. TriNetX adheres to the Health Insurance Portability and Accountability Act (HIPAA) standards to ensure that all patient-level and aggregated data are de-identified, following the guidelines of the HIPAA Privacy Rule, particularly Section §164.514(a)^10^. The de-identification process is validated by a qualified expert as required by Section §164.514(b) of the HIPAA Privacy Rule, negating the need for a prior waiver from the Western Institutional Review Board (IRB). 128 Healthcare Organisations who contributed de-identified data to TriNetX included academic university hospitals, community hospitals, and specialty physician services covering inpatient and outpatient care.

To address baseline imbalances, we applied propensity score matching (PSM) using a 1:1 greedy nearest neighbor matching algorithm with a caliper of 0.1 standard mean difference to ensure precise matching. We adjusted for covariates, including demographic factors including age, gender, race, and common cardiovascular risk factors like hypertension and diabetes mellitus. Propensity scores were calculated by bivariate logistic regression, including the following variables that might be considered as potential baseline confounders between the groups: sex, age and race. We matched propensity scores 1:1 with the use of the nearest neighbor methods without replacement by using the closest caliper width to achieve the maximum number of cases without statistical differences in confounding variables. Following matching, both groups comprised 26,160 patients with comparable baseline characteristics.

Our primary objective was to assess the impact of cirrhosis on 1-year gastrointestinal and nutritional outcomes in AP patients, focusing on pseudocyst formation, protein-calorie malnutrition, ileus, small bowel obstruction and total parenteral nutrition. The outcomes were queried using the relevant ICD-10 codes.

### Statistical analysis

The analyses were conducted using TriNetX’s integrated analytical tools, which utilize R and SQL-based backend analytics within a secure cloud environment. Descriptive statistics summarized demographic and clinical characteristics and compared between groups using the student t-test or Mann-Whitney U test for continuous variables and chi-square or Fisher’s exact test for categorical variables. Kaplan-Meier survival analysis assessed risks between cohorts by comparing cirrhosis cohorts and non-cirrhosis cohorts using the log-rank test and calculating hazard ratios (HRs). All statistical tests were two-sided, and statistical significance was defined as a *P* value of <0.05.

## RESULTS

Prior to Propensity score matching, the Acute pancreatitis cohort with cirrhosis (cohort 1) of 27,001 patients had a mean age of 56.1 years and was predominantly white (62.4%) with a lower proportion of females (40.1%) and American Indian or Alaska Native (1.1%), a higher proportion of Native Hawaiian or Pacific Islander (1.1%), Hispanic or Latino (12%), Black or African American (13.5%), Males (56.8%) and Asians (5.6%). In comparison, the AP cohort without cirrhosis (cohort 2) of 592,833 patients had a mean age of 53.3 years, fewer whites (57.3%) and a higher proportion of females (49.2%)., with less American Indian or Alaska Native (0.5%), Native Hawaiian or Pacific Islander (0.8%), Hispanic or Latino (8.6%), Black or African American (11.9%), Males (48.4%) and Asians (3.7%).

**FIGURE 1:**
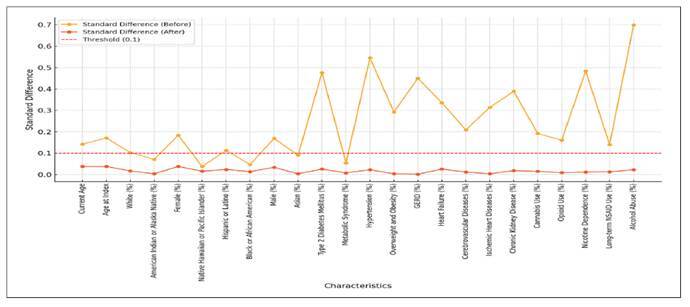
Standad differences befre and after matching by characteristic.

Significant differences were also noted in the prevalence of several comorbidities, including Type 2 diabetes mellitus, metabolic syndrome, hypertension, overweight and obesity, GERD, heart failure, cerebrovascular diseases, ischaemic heart diseases, chronic kidney diseases, cannabis use, opioid use, nicotine dependence, long term NSAID use, alcohol abuse. After propensity score matching (PSM), both cohorts had 26,160 patients with similar mean ages (56.1 vs 56.6) years respectively (Table 1 WITH Figure 1).

**TABLE 1:** Baseline characteristics of patients with acute pancreatitis (AP) stratified into two cohorts: Cohort 1 includes AP patients with cirrhosis, and Cohort 2 includes AP patients without cirrhosis before and after propensity Matching.

Characteristic	Before Matching (Cohort 1 vs Cohort 2)	Standard Difference (Before)	After Matching (Cohort 1 vs Cohort 2)	Standard Difference (After)
**Demographics**				
Current Age (years)	62.1±13.9 vs 59.8±18.4	0.142	62.2±13.9 vs 62.7±15.4	0.038
Age at Index (years)	56.1±13.7 vs 53.3±18.6	0.171	56.1±13.7 vs 56.6±15.4	0.038
White (%)	62.4% vs 57.3%	0.103	62.4% vs 63.2%	0.017
American Indian or Alaska Native (%)	1.1% vs 0.5%	0.071	1.1% vs 1.2%	0.004
Female (%)	40.1% vs 49.2%	0.184	40.1% vs 38.3%	0.038
Native Hawaiian or Pacific Islander (%)	1.1% vs 0.8%	0.038	1.1% vs 1.3%	0.016
Hispanic or Latino (%)	12.0% vs 8.6%	0.113	12.0% vs 11.2%	0.024
Black or African American (%)	13.5% vs 11.9%	0.047	13.5% vs 14.0%	0.014
Male (%)	56.8% vs 48.4%	0.169	56.8% vs 58.5%	0.034
Asian (%)	5.6% vs 3.7%	0.091	5.6% vs 5.7%	0.004
**Comorbidities**				
Type 2 Diabetes Mellitus (%)	36.8% vs 16.3%	0.476	36.7% vs 38.0%	0.026
Metabolic Syndrome (%)	0.4% vs 0.1%	0.054	0.4% vs 0.4%	0.008
Hypertension (%)	57.0% vs 30.8%	0.546	56.9% vs 58.1%	0.023
Overweight and Obesity (%)	23.6% vs 12.5%	0.292	23.5% vs 23.4%	0.004
GERD (%)	37.2% vs 17.6%	0.45	37.2% vs 37.1%	0.002
Heart Failure (%)	16.7% vs 6.2%	0.336	16.7% vs 15.8%	0.026
Cerebrovascular Diseases (%)	12.6% vs 6.5%	0.209	12.6% vs 12.2%	0.012
Ischemic Heart Diseases (%)	23.7% vs 11.9%	0.314	23.7% vs 23.5%	0.004
Chronic Kidney Disease (%)	21.6% vs 8.0%	0.389	21.6% vs 20.8%	0.019
Cannabis Use (%)	4.3% vs 1.2%	0.192	4.3% vs 4.0%	0.015
Opioid Use (%)	2.6% vs 0.6%	0.161	2.6% vs 2.5%	0.009
Nicotine Dependence (%)	31.0% vs 11.7%	0.484	31.0% vs 31.5%	0.012
Long-term NSAID Use (%)	2.9% vs 1.0%	0.14	2.9% vs 2.7%	0.011
Alcohol Abuse (%)	29.8% vs 4.8%	0.699	29.8% vs 28.7%	0.023

In a matched cohort of 26,160 cirrhosis and 26,160 non cirrhotic patients, the most pronounced difference was seen in protein calorie malnutrition (1360.32 vs 680.16; HR 2.070). Across all gastrointestinal and nutritional outcomes, patients with cirrhosis experienced consistently higher event rates except in pseudocyst formation.

**FIGURE 2:**
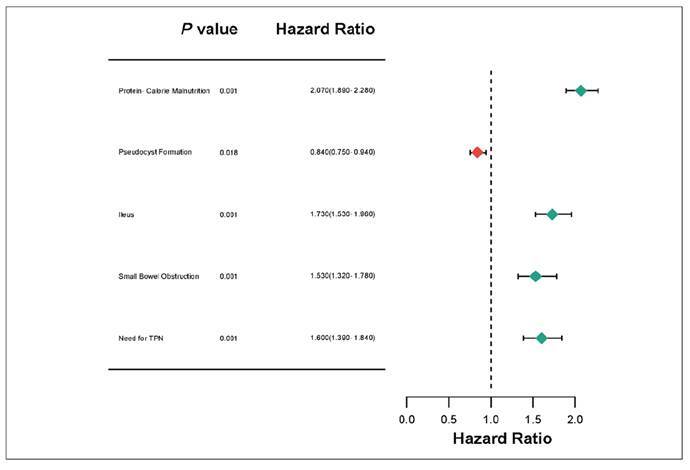
Forest plot depicting hazard ratios (HR) and 95% confidence intervals (CI) for 1-year outcomes in acute pancreatitis (AP) patients with and without cirrhosis. The plot highlights significant associations between cirrhosis and increased risks for nutritional deficiencies.

Moderate increase was seen in ileus (706.32, HR 1.730), Small bowel obstruction (444.72, HR:1.530), need for TPN (497.04 vs 313.92, HR 1.600), with decreased rate in pseudocyst formation (601.68 vs 732.48, HR 0.840). PEG insertion and abdominal compartment syndrome showed no significant differences. These outcomes indicate an attributable outcome of these complications in Acute pancreatitis patients with cirrhosis (Figure 2).

## DISCUSSION

Acute pancreatitis (AP) is a critical gastrointestinal condition with increasing global incidence and significant burden of morbidity and mortality^11^. This retrospective cohort study aimed to evaluate the attributable risk of liver cirrhosis (LC) on the nutritional and gastrointestinal outcomes in patients with AP. Our findings indicate that cirrhosis significantly worsens both nutritional status and gastrointestinal outcomes in this population except for pseudocyst formation which was found to have a decreased incidence. This highlights a compounded clinical burden and emphasizes the need for more tailored management strategies.

While extensive literature exists on the individual complications of cirrhosis and AP, few studies have focused on their combined impact, particularly on nutrition and gastrointestinal function. This retrospective cohort study emphasizes that the coexistence of LC in patients with AP results in higher risks of protein-calorie malnutrition, ileus, small bowel obstruction and need for TPN but interestingly, decreased pseudocyst formation.

Protein-calorie malnutrition was found to be more prevalent in patients with both AP and LC according to our study. This heightened risk can be attributed to multiple factors: In AP, there may be increased catabolic stress, anorexia due to abdominal pain, and inflammatory responses leading to elevated metabolic demands^12^ ^,^ ^13^. Cirrhosis adds a further nutritional burden through mechanisms such as early satiety from ascites, poor appetite from dietary sodium restriction, altered taste from micronutrient deficiencies (e.g., zinc, magnesium), and impaired fat absorption due to reduced bile production^14^. Additionally, hepatic insulin resistance, increased energy expenditure, and accelerated gluconeogenesis contribute to muscle wasting and progressive malnutrition in cirrhotic patients^15^ ^-^ ^17^.

According to Teran (1999), Malnutrition in cirrhosis is well documented, with prevalence rates ranging from 34-82% in patients with alcoholic cirrhosis and 27-87% in patients with non-alcoholic cirrhosis, varying based on assessment method. In a study by Khadka et al. (2019) on 54 cirrhosis patients, malnutrition was observed in up to 66.7% based on handgrip strength, 40.74% based on mid upper arm muscle circumference and 59.26% according to triceps skin fold thickness further illustrating the nutritional vulnerability in this cohort. Importantly, alcohol a common etiological factor for both cirrhosis and AP exacerbates nutritional deficiencies and impairs gastrointestinal integrity^18^ ^,^ ^19^.

Current guidelines, such as those from the European Society for Clinical Nutrition and Metabolism (ESPEN), recommend that all patients with AP undergo nutritional screening, with those having severe disease presumed to be at high risk^18^ ^,^ ^20^. Our findings support this recommendation and further advocate for routine, early nutritional assessment in AP patients with comorbid cirrhosis, regardless of disease severity, due to their dual nutritional risk.

Our study observed an increased rate of Total Parenteral Nutrition (TPN) usage in patients with both cirrhosis and acute pancreatitis, highlighting a critical aspect of their nutritional management. In general AP populations, early oral feeding and enteral nutrition (EN) are preferred, particularly in mild to moderate cases, due to their gut-protective effects and lower risk of infectious complications. Guidelines recommend initiating EN as soon as tolerated to preserve mucosal integrity, reduce bacterial translocation, and avoid the complications associated with parenteral nutrition. However, in severe cases such as those with abdominal compartment syndrome, persistent paralytic ileus, intestinal obstruction, or intolerance to EN, TPN becomes a necessary alternative to ensure adequate caloric and protein intake^18^.

In cirrhotic patients with AP, nutritional management becomes more complex due to concurrent challenges such as ascites, gastrointestinal dysmotility, and impaired nutrient absorption^5^ ^,^ ^21^ ^,^ ^22^. Simons-Linares et al. (2020) reports that cirrhotic patients often present with fluid overload, making aggressive intravenous (IV) fluid resuscitation the mainstay of early AP management difficult to administer safely. Acute pancreatitis induces capillary leak and third-spacing, which leads to hypovolemia, hypotension, and potential multi-organ failure if not rapidly corrected. However, in cirrhotic patients particularly those with decompensated disease, fluid resuscitation must be carefully balanced to avoid worsening ascites, pleural effusions, and volume overload^5^.

This fluid management dilemma may further predispose cirrhotic AP patients to inadequate perfusion and gut ischemia, thereby increasing EN intolerance and shifting nutritional support toward TPN. Currently, guidelines do not adequately address whether standard aggressive fluid protocols (e.g., 250-500 mL/hour) are appropriate or harmful in patients with cirrhosis^5^. Future prospective studies are urgently needed to determine optimal fluid resuscitation strategies in this vulnerable population and to establish evidence-based nutritional guidelines tailored to cirrhotic individuals with AP. Until such data are available, individualized care with close hemodynamic monitoring, cautious fluid management, and early involvement of nutrition specialists is essential.

Gastrointestinal complications were also more pronounced in AP patients with LC. The development of ileus, for instance, was significantly more frequent in cirrhotic individuals with AP. Several pathophysiological mechanisms such as hypoalbuminemia, systemic inflammation, opioid analgesia, and reduced mobility may contribute to ileus in these patients^23^ ^-^ ^25^. In a large cohort study conducted by Singh et al the presence of chronic liver disease was associated with a higher incidence of clinically significant ileus in patients with AP (24.1% vs 20.63%). A study by Simons Linares et al. also reported increased incidence of ileus in cirrhotic patients with AP (OR: 1.3, *P*=0.02).

Small bowel obstruction (SBO), though less frequently discussed in the context of acute pancreatitis, is an emerging and clinically significant complication, though rare that may be seen patients with AP patients. Case reports and small observational studies have described SBO may result from mechanisms that similarly cause mechanical bowel obstruction including pancreatic inflammation causing extrinsic compression, retroperitoneal inflammation, enzyme extravasation, and fibrosis affecting small intestine^26^. Cirrhosis may exacerbate this risk through altered intestinal motility, vascular congestion, and increased intestinal wall edema due to portal hypertension. Gunnarsdottir et al. also reported that patients with cirrhosis commonly experience small intestinal dysmotility and are predisposed to small intestinal bacterial overgrowth (SIBO), which further complicates gastrointestinal function^22^. Though data remain limited, the effects of inflammatory, structural, and motility-related insults make SBO a plausible and underrecognized complication in cirrhotic patients with AP.

A particularly notable aspect of our study was the decreased incidence of pancreatic pseudocyst (PC) formation in patients with cirrhosis (2.3%) compared to non-cirrhotic patients (2.8%). This contrasts with findings from Hoferica et al., who reported increased odds of pancreatic complications including acute necrotic collections (ANC), acute peripancreatic fluid collections (APFC), and pancreatic pseudocyst formation in cirrhotic patients. Given these discrepancies, the relationship between cirrhosis and pseudocyst development remains unclear and warrants further investigation.

One possible explanation for our observed reduction in pseudocyst formation may lie in the pathophysiology of cirrhosis-associated immune dysfunction (CAID). Pseudocyst formation typically requires a prolonged and coordinated inflammatory response following the persistence of peripancreatic fluid collections, often evolving over 4 to 8 weeks^27^. In patients with advanced cirrhosis, however, immune function becomes increasingly impaired. As CAID progresses from an early pro-inflammatory state to one of immune paralysis in severe or decompensated cirrhosis, monocyte and neutrophil activity becomes diminished, chemotaxis and phagocytosis are impaired, and proinflammatory cytokines such as IL-1 and TNF-α are suppressed in favor of elevated anti-inflammatory cytokines like IL-6 and IL-10^8^ ^,^ ^28^. This attenuated immune response may blunt the inflammatory cascade necessary for pseudocyst encapsulation, potentially explaining the reduced incidence observed in our cohort.

Also as mentioned earlier Pseudocyst formation typically develops over weeks after acute pancreatitis^27^. Cirrhotic patients with severe disease may not survive long enough or may have altered healing responses that affect cyst formation.

In summary, this study demonstrates that cirrhosis independently contributes to worsened nutritional and gastrointestinal outcomes in acute pancreatitis, serving as a significant modifier of disease course. The attributable risks include heightened malnutrition, increased likelihood of gastrointestinal dysmotility (e.g., ileus), greater susceptibility to bowel obstruction and greater need for TPN, with a possible decreased risk of pseudocyst formation. Clinicians should recognize cirrhotic AP patients as a high-risk subgroup requiring early nutritional intervention, vigilant gastrointestinal monitoring, and individualized supportive care. Future studies should aim to quantify these risks more precisely and inform clinical guidelines on integrated management strategies for patients with both conditions.

### Limitations

In designing this study, our primary objective was to evaluate whether cirrhosis, as a broad clinical entity, is associated with worse outcomes in patients with acute pancreatitis. While the TriNetX platform has the capacity for stratification by cirrhosis etiology (e.g., alcoholic vs non-alcoholic) or pancreatitis severity, it becomes technically challenging to apply such extensive sub-stratification at the stage of building matched cohorts without compromising statistical balance and comparability. For this reason, we did not subdivide patients according to Child-Pugh or MELD scores, fibrosis stage, or cirrhosis etiology, but instead treated cirrhosis as a unified variable. Further exploration of cirrhosis staging, pancreatitis etiology/severity, and body composition parameters would add valuable context in future studies.

Also, the severity of acute pancreatitis was not graded, limiting our ability to adjust for disease severity as a confounder. Data on nutritional status were derived from coded diagnoses and treatment patterns (e.g., TPN use), which may underestimate subclinical malnutrition. Residual confounding from unmeasured variables such as alcohol intake, socioeconomic status, and medication use (e.g., diuretics, beta-blockers) cannot be excluded. Survival bias may also have influenced the lower incidence of pseudocyst formation observed in cirrhotic patients, as those with advanced disease may not have survived long enough for this complication to develop.

Additionally, the paucity of literature focusing specifically on the intersection of cirrhosis and acute pancreatitis in relation to gastrointestinal and nutritional outcomes posed a challenge in contextualizing our findings. Most available evidence addressed these conditions in isolation, or grouped cirrhotic patients with other chronic comorbidities, thereby diluting specific attributable risks. Furthermore, nutritional assessments varied widely across studies, with different anthropometric and biochemical measures, which may impact the consistency of reported malnutrition prevalence. Finally, as this was a single-database study, the findings may not be generalizable across all healthcare settings.

### Implications for practice

The findings from this review have several important implications for clinical practice:

Risk stratification: clinicians should proactively identify patients with concurrent cirrhosis and acute pancreatitis as a distinct high-risk subgroup. Early identification enables closer monitoring, risk mitigation, and improved allocation of multidisciplinary care.

Early nutritional assessment and intervention: given the significantly elevated risk of protein-calorie malnutrition in cirrhotic AP patients, early and standardized nutritional screening using validated tools is critical. Where feasible, initiating enteral nutrition early in the course of AP should be prioritized to prevent deterioration.

Tailored gastrointestinal monitoring: the increased risk of ileus and small bowel obstruction necessitates vigilant gastrointestinal monitoring. This includes careful fluid management, avoidance of unnecessary opioid use, and timely imaging in the presence of persistent abdominal symptoms.

Integrated multidisciplinary approach: optimal care for these patients should involve a coordinated team including gastroenterologists, hepatologists, dietitians, and critical care specialists. Individualized care plans that account for hepatic dysfunction, nutritional needs, and gastrointestinal complications are essential.

Clinical guidelines development: there is a need to develop targeted clinical guidelines that address the dual pathology of cirrhosis and AP, especially concerning nutritional management, gastrointestinal surveillance, and complication prevention.

## CONCLUSION

This study demonstrates that liver cirrhosis significantly worsens both nutritional and gastrointestinal outcomes in patients with acute pancreatitis, identifying it as an independent and clinically relevant risk factor. Cirrhotic patients with AP exhibited higher rates of protein-calorie malnutrition, gastrointestinal complications such as ileus and small bowel obstruction, and increased need for total parenteral nutrition due to intolerance of enteral feeding.

Interestingly, our findings also revealed a decreased incidence of pancreatic pseudocyst formation in cirrhotic patients, suggesting a potential modification in the typical local complications associated with acute pancreatitis in the context of chronic liver disease. The underlying reasons for this observation are not yet fully understood and warrant further investigation.

The interplay between cirrhosis and AP poses unique challenges, particularly in fluid resuscitation and nutritional support, areas where standardized guidelines remain lacking. Given the rising prevalence of both conditions, there is a pressing need for targeted clinical protocols that address the specific risks and management needs of this high-risk population. Future prospective studies should aim to quantify the attributable risk more precisely and guide evidence-based strategies to improve outcomes in patients with cirrhosis and acute pancreatitis.

## Data Availability

Research data are presented within the article itself (Table 1 & Figure 1).
